# Mental Training for Better Achievement: Effects of Verbal Suggestions and Evaluation (of Effectiveness) on Cognitive Performance

**DOI:** 10.3389/fpsyt.2018.00510

**Published:** 2018-10-22

**Authors:** Kristina Fuhr, Dustin Werle

**Affiliations:** Clinical Psychology and Psychotherapy, Department of Psychology, University of Tuebingen, Tuebingen, Germany

**Keywords:** placebo, mental training, cognitive performance, verbal suggestions, effectiveness rating

## Abstract

**Objective:** There is only some literature regarding the influence of verbal suggestions on cognitive performance in healthy volunteers. For example, the performance in a knowledge test was enhanced when participants were told that they had subliminally received the correct answer. However, enhancing cognitive performance only via verbal suggestions without prior conditioning phases has not yet been examined. The goal of our study was therefore to investigate the effects of a mental training based on verbal suggestions compared to a control training on cognitive performance in a student population using a balanced-placebo-design.

**Methods:** In total, 103 participants were randomly assigned either to listening to a 20 min audio-taped mental training or to a 20 min philosophy lecture (control training) via headphones. Participants were individually tested before and after the training concerning their cognitive performance. Information about the type of training were varied in both intervention conditions (“You are part of our experimental condition and you will receive an effective mental training” or “You are part of our control group and you will receive the control condition”). At the end of the assessment, participants were asked what kind of training they believed they had received and how effective they would rate the received training.

**Results:** Overall, the cognitive performance improved in all participants, *F*_(1, 99)_ = 490.01, *p* < 0.001. Contrary to our hypotheses, we found no interaction of the type of training and type of instruction on the cognitive performance. Participants who rated the received training as being effective at the end of the experiment (regardless if it was the mental or the control training), have before experienced a greater improvement in their cognitive performance [*F*_(2,100)_ = 7.26, *p* = 0.001] and showed higher scores in the ability to absorb [*F*_(2, 99)_ = 3.75, *p* = 0.027].

**Conclusion:** The subjects' own experiences in the task might have influenced the rating of the training rather than the actual training or the information they receive regarding the type of training. This finding underlines the relevance of enhancing the subjective beliefs and self-efficacy in situations where cognitive attention processes are important and of individually tailoring mental trainings.

## Introduction

The influence of expectations and suggestions on the placebo response was shown in different experimental studies concerning psychological aspects like for example pain [e.g., (1)]. In the context of pain, the given verbal instructions did not only influence the subjective analgesic effect but also reduced the amount of requests for opioid doses (1). However, verbal suggestions alone (without any preconditioning) could only influence pain tolerance in healthy subjects and motor performance in Parkinson's patients, but not for example hormonal secretion (2). Little is known about placebo effects on cognitive performance. For example, cognitive processes like attention could be improved after suggesting participants they had consumed caffeine similar to the improvement after they really consumed caffeine [e.g., (3)]. Comparable results were found when college students believed that they had received a “neuro-enhancing” stimulant. The cognitive performance improved in some scales and the participants also evaluated their subjective results as better (4). However, the placebo effect concerning the cognitive processes was only investigated when providing the participants with some kind of substance or placebo. Therefore, it would be interesting if similar to the results that were found in pain, cognitive processes might also be influenced by evoking specific expectations just via receiving verbal suggestions, i.e., verbal instructions but also via psychological interventions. Automatic visual perception and cognitive processes, as they are for example assumed for the Stroop effect, can be influenced and even controlled just by receiving verbal suggestions during a hypnotic experience. This effect was most pronounced in highly suggestible individuals (5, 6). For example, the interference in the Stroop effect, when the ink color and the word color are incongruent, could be eliminated when suggesting subjects to view word stimuli as neutral and meaningless (5). Furthermore, the performance of counting visual stimuli was reduced when suggesting participants after a hypnotic induction that a wooden board would cover the screen (6). In one study, the performance in the Flanker task, for example, was only influenced by posthypnotic suggestions in highly suggestible participants compared to (the same) non-hypnotic suggestions in an alert state (7). Thus, the advantage of using verbal suggestions after a hypnotic induction or with highly suggestible participants instead of only verbal instructions is that hypnotic suggestions were able to even demonstrate control over some cognitive processes, as described before (5, 6). However, there is only some literature regarding the enhancement of cognitive performance only via verbal suggestions. For example, the performance in a knowledge test was enhanced when participants believed that they had subliminally received the correct answer (placebo condition) compared to those who were told that they subliminally only received a flash [control condition, (8)]. Even the results in an intelligence test could be enhanced when positive expectations were evolved only via the way participants were recruited (9). Another study found that it was more the subjective evaluation of the own performance that was influenced by expectations rather than the objective performance [as for example reaction times or success rates, see (10)]. However, in previous studies, expectancy effects on placebo were usually paired with some previous test phase in which participants already underwent a specific conditioning paradigm and therefore could already build according expectations regarding the relevant test phase (10).

Taken together, previous results imply that some suggestions can block cognitive processes that were assumed to be automatic and not directly influenceable. However, the magnitude of the placebo effect on enhancing cognitive performance only based on verbal suggestions without previous experience has not yet been examined.

Concerning placebo effects in psychotherapy, as in well-established treatments like for example cognitive behavioral therapy, it is impossible to conduct double-blind trials and challenging to develop “placebo” control groups that are not distinguishable from the specific treatment that is to be tested (11). However, it was demonstrated that psychological placebo interventions show equivalent effects as specific psychotherapies if they were structurally equally designed (12). That's why some researchers emphasize the relevance/superiority of the “common factors” in psychotherapy over the specific therapeutic ingredients (13, 14). Investigating mechanisms of change and differentiating specific and non-specific/common therapeutic “ingredients” is more or less impossible since every specific ingredient is transmitted also via words, verbal suggestions, and other therapeutic rituals (15). Studies are lacking that directly manipulate some of the non-specified factors as for example expectations (16). Using hypnotic verbal suggestions could be one possibility to use some kind of psychological intervention that is not based on specific treatment strategies but directly addresses the non-specific factors like expectations (17) and hypnosis can thus be used as a “non-deceptive placebo” (18).

The goal of our study was therefore to investigate the effects of a mental training based on hypnotic verbal suggestions compared to a control training consisting of a philosophy lecture on the cognitive performance in a student population using a “balanced-placebo-design” (19). We paired the trainings with evoking different expectations in the participants concerning the “efficacy” of the mental training. We expected the strongest effects on cognitive performance when participants received both the mental training and the suggestion of this training as being “effective” compared to the conditions in which they received incongruent information (received mental training and were told “ineffective” or received control training and were told “effective”) or the control training paired with the information of being “ineffective.”

## Materials and methods

### Study design

**Figure 1:**
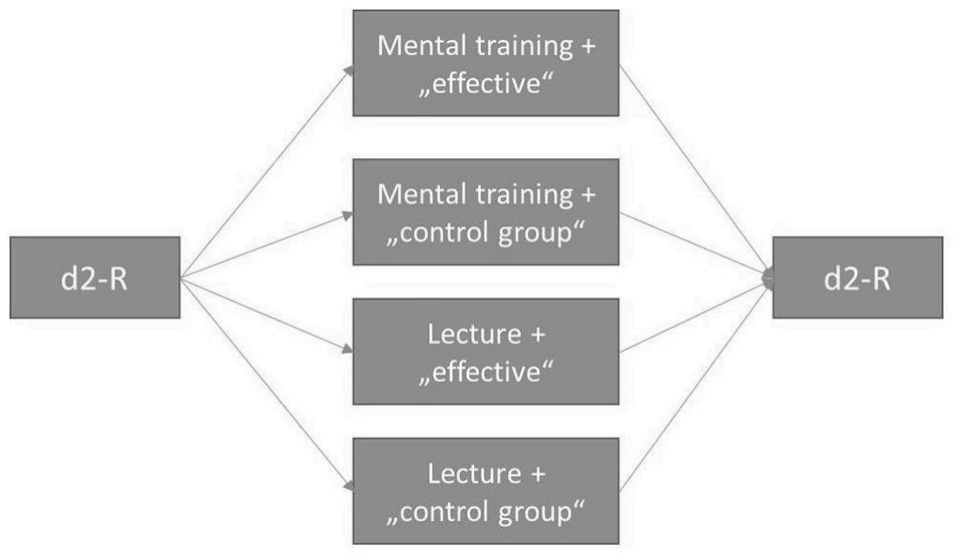
Study design and procedure.

The study used a mixed 2 × 2 × 2 design with the within-factor time and the between-factors intervention and information, see Figure 1. As dependent variable, we assessed the cognitive performance in a specific attention/concentration task (see Assessments). With the within-factor time, cognitive performance was measured before and after the intervention. The factor intervention consisted of the mental training vs. the control training. Concerning the factor information, half of the participants of each intervention condition was told to receive an effective training (“You are part of our experimental group and you will receive an effective mental training”) vs. the other half was told that they are part of the control group (“You are part of our control group and you will receive the control condition”). With pairing the factors intervention and information, a balanced-placebo-design was established. Subjects were randomly assigned to one of the four experimental conditions. We chose random numbers between one to four that were equally distributed to do so (source: https://rechneronline.de/zufallszahlen/).

The study protocol was approved by the local Ethics Committee for Psychological Research of the Faculty of Science at the University of Tuebingen (Az 2016/1123/26). All subjects gave written informed consent in accordance with the Declaration of Helsinki.

### Participants

The a priori sample size was defined at 100 participants.

Participants for the current study were recruited at the University of Tuebingen via announcements in social media (e.g., facebook groups of psychology students and of the students who were interested in study participation) and in cafeterias and libraries. Inclusion criteria were (1) being at the age of 18 to 50 years old, (2) no uncorrected ametropia, (3) normal hearing, and (4) providing written informed consent to participate in the study. Participants were alerted to the fact that they might be incompletely informed about some part of the study [“authorized deception,” see (20)]. After study completion, every participant was completely debriefed in case of prior deception. The “misinformation” of participants was necessary in these experimental conditions where the type of intervention and the type of information were not congruent (mental training and told that “being part of the control group”; control training and told that “being part of the experimental group and receiving an effective training”). The goal of the incomplete information was to evoke specific expectations in participants concerning the effectiveness of the following intervention. The so-called “authorized deception” is a possibility in experimental placebo studies to overcome an important ethical dilemma. On one hand, the deception of participants is necessary to demonstrate the effect of expectancy on outcome. On the other hand, the ethical norms request the participant's free choice of taking part in a study only after every aspect of the study was fully displayed (21). It was shown that the use of “authorized deception” does not affect the placebo effect and therefore is a very useful tool (20).

In total, 103 participants were investigated in the present study. Eighty-three of the subjects (80.6%) were female, all of them being students, about 50% were studying psychology (*n* = 43, 41.7%) or cognitive science (*n* = 10, 9.7%) in the first or second year. The mean age was 23.35 years (*SD* = 4.18).

### Interventions

In both conditions, participants individually listened to a 20 min audio-take via headphones. The mental training consisted of different indirect hypnotic verbal suggestions for enhancing the cognitive performance: reminding participants of their own experience when learning some new procedures, creating the image of an archer, giving metaphors with the goal to concentrate and focus on relevant aspects of a task whereas irrelevant aspects can be ignored. Participants were thus not directly but indirectly told to focus more on the cognitive performance task. The control training consisted of a part of a lecture in philosophy about the humans' free will. Listening to the lecture should not evoke an enhancement of the cognitive performance in the subjects. The “control training” was parallelized in the length to the mental training.

### Assessments

The cognitive performance of participants was individually tested with the d2R (22) before and after receiving the intervention. The d2R measures attention and concentration performance within 5 min. Participants have to cancel out every “d” with two dashes in between distractors across several rows within a specific time limit. We used the measure of concentration performance of the d2R as overall measure for the participants' attention ability.

Depressive symptoms within the last 14 days were assessed with the Beck Depression Inventory II at baseline [BDI II, (23)].

The participants' ability to absorb in thoughts and imaginings was assessed with the Tellegen Absorption Scale with 34 items at baseline [TAS, (24)].

The BDI-II and the TAS were included as covariates in the data analysis.

At baseline, we also asked participants via questionnaire about some sociodemographic variables.

At the end of the assessment, participants were asked to rate (1) the effectiveness of the training and (2) what type of training the believed they received.

### Study procedure

The investigators of the study followed a specific protocol with standardized instructions when interacting with the participants. Investigators were not blinded to the intervention and the information condition of the participants. After oral and written informed consent, participants received the BDI-II, the TAS, and a sociodemographic questionnaire. At baseline, the d2R was administered the first time. Afterwards, information about the type of training was varied according the participant's experimental condition (“You are part of our experimental condition and you will receive an effective mental training” or “You are part of our control group and you will receive the control condition”). Afterwards, participants received either the mental training or the philosophy lecture via headphones for 20 min. After listening to the audios, the participants were again tested with the d2R. After the task, they were asked to answer the previously described questions about the training they have received before. At the end of the assessment, all participants were debriefed following again a standardized protocol. The assessment took 1 h in total per participant. Subjects received either monetary compensation or got hourly credit for participating in the study.

### Statistical analysis

We computed a three-way analysis of variance, with the between-subjects factors intervention (mental training vs. control training) and information (“effective training” vs. “part of the control group”), and the within-subjects factor time (before and after the training). The dependent variable was cognitive performance measured before and after receiving the training. For baseline correction, we conducted an analysis of covariance with the factors intervention and information, as well as cognitive performance at baseline as covariate. Cognitive performance after the training was used as dependent variable.

## Results

The four study groups were comparable at baseline regarding age, *F*_(3, 99)_ = 0.21, *p* = 0.890, sex, *X*^2^_(3)_ = 3.79, *p* = 0.285, the TAS, *F*_(3, 99)_ = 0.56, *p* = 0.644, and the BDI-II, *F*_(3, 99)_ = 0.41, *p* = 0.748.

There were differences in the four groups in the cognitive performance at baseline, almost reaching significance, *F*_(3, 99)_ = 2.61, *p* = 0.056, see also the following analyses.

The cognitive performance significantly improved in all participants, *F*_(1, 99)_ = 490.01, *p* < 0.001. Means and standard deviations are displayed in Table 1. We found no effect of the type of intervention, *F*_(1, 99)_ = 0.11, *p* = 0.747, and no interaction between the factors intervention and time, *F*_(1, 99)_ = 0.06, *p* = 0.802. Further, we found no interaction between information and time, *F*_(1, 99)_ = 0.71, *p* = 0.402, and between information and intervention, *F*_(1, 99)_ = 0.85, *p* = 0.358. However, we found a significant interaction between all three factors, *F*_(1, 99)_ = 4.08, *p* = 0.046 and a significant effect of type of information, *F*_(1, 99)_ = 4.79, *p* = 0.031. This was due to significant differences in the cognitive performance at baseline regarding the type of information, *F*_(1, 101)_ = 5.72, *p* = 0.018. The participants that were later told to be in the control group were actually faster than those who were later instructed to receive an effective mental training (“effective mental training”: *M* = 166.4, *SD* = 34.8 vs. “control group”: *M* = 183.1, *SD* = 36.1).

**Table 1:** Cognitive performance before and after the intervention in all four experimental conditions.

**Intervention**	**Information**	**d2-R KL pre *M (SD)***	**d2-R KL post *M (SD)***
Mental training	“Effective mental training” (*n* = 29)	169.8 (32.8)	205.3 (36.1)
	“Control group” (*n* = 24)	176.7 (32.4)	216.3 (36.5)
Lecture (Control training)	“Effective mental training” (*n* = 25)	162.6 (37.3)	204.1 (39.0)
	“Control group” (*n* = 25)	189.3 (39.0)	221.2 (40.5)
	total (*n* = 103)	174.4 (36.2)	211.4 (38.1)

*M, mean; SD, standard deviation; n, sample size*.

When controlling for baseline differences in cognitive performance, there was an interaction between type of training and type of instruction, almost reaching significance, *t*_(98)_ = −12.77, *p* = 0.063. The cognitive performance in groups with congruent information and training did not improve as much, as in groups with incongruent information (improvement “congruent”: *M* = 33.89, *SD* = 20.11 vs. improvement “incongruent”: *M* = 40.55, *SD* = 12.38).

The covariate depressive symptoms, assessed with the BDI-II, was not related to the cognitive performance, *F*_(1, 96)_ = 0.13, *p* = 0.723, nor was the ability to absorb, as measured with the TAS, *F*_(1, 96)_ = 0.46, *p* = 0.500.

When we compared the cognitive performance of the participants regarding their effectiveness rating at the end of the intervention, a significant interaction between time and the rating of the training was found. We observed an improvement of the cognitive performance, when the participants afterwards rated the training as neutral or effective at the end of the assessment, compared to those who rated the training as ineffective, *F*_(2, 100)_ = 7.26, *p* = 0.001, see also Table 2. The improvement in cognitive performance was, however, independent of the fact if participants correctly identified the training condition or not, *F*_(1, 101)_ = 1.75, *p* = 0.189.

**Table 2:** Cognitive performance before and after the intervention regarding effectiveness ratings.

	**d2-R KL pre *M (SD)***	**d2-R KL post *M (SD)***
Training was ineffective (*n* = 24)	179.71 (36.45)	205.75 (34.18)
Training was neutral (*n* = 29)	171.90 (32.55)	212.59 (38.65)
Training was effective (*n* = 50)	173.26 (38.56)	213.50 (40.02)

*M, mean; SD, standard deviation; n, sample size*.

The actual training they received had no significant effect on the evaluation of the effectiveness, *X*^2^_(2)_ = 5.50, *p* = 0.064, see Table 3. The effectiveness rating at the end of the intervention was rather significantly associated with their own belief, what type of training they received, *X*^2^_(2)_ = 10.67, *p* = 0.005, see Table 4.

**Table 3:** Effectiveness rating in the two actual intervention conditions.

	**Mental training: *n***	**Lecture: *n***
Training was ineffective (*n* = 24)	8	16
Training was neutral (*n* = 29)	14	15
Training was effective (*n* = 50)	31	19

*n, sample size, X^2^_(2)_ = 5.50, p = 0.064*.

**Table 4:** Effectiveness rating in the two assumed intervention conditions.

	**Mental training assumed: *n***	**Lecture assumed: *n***
Training was ineffective (*n* = 24)	8	16
Training was neutral (*n* = 29)	8	21
Training was effective (*n* = 50)	31	19

*n, sample size, X^2^_(2)_ = 10.67, p = 0.005*.

These participants who rated the training as neutral or effective, showed higher scores in absorption (TAS) at baseline compared to those who rated the training as ineffective, *F*_(2, 99)_ = 3.75, *p* = 0.027 (ineffective: *M* = 42.0, *SD* = 19.6; neutral: *M* = 46.4, *SD* = 18.3; effective: *M* = 56.1, *SD* = 25.4; *post hoc* Bonferroni: effective vs. ineffective: *p* = 0.038). No differences were found regarding age, depressive symptoms, and sex.

## Discussion

Contrary to our hypothesis, that a mental training, which is only based on verbal suggestions, or the information about the effectiveness of the training could enhance cognitive performance, there was no effect of the training and the information about the training on their actual performance. Overall, we found that the cognitive performance of all participants improved. This result might be due to practicing or an effect of repeated measures [see also (22)]. However, the participant's own rating about the effectiveness of the training was significantly influenced by the cognitive performance change irrespective of the actual type of intervention or information they received. Our results suggest that it was either the perception of their own performance improvement that influenced their rating about the effectiveness at the end of the assessment. The effectiveness rating, however, was independent of the actual training they received and also of the fact if they correctly identified the training condition. Thus, another interpretation of the results could be that the subjective evaluation influenced the participant's performance in the second trial. Unfortunately, we did not assess expectations regarding the mental training and its influence on cognitive performance before the assessment.

Mental practice is known to enhance performance in general, because it involves training of specific behaviors, especially in cognitive tasks (25). Another study found that mental practicing - imagining a specific motor activity—could enhance the outcome in that specific motor task (26). This finding could be interpreted as a top-down mechanism that somehow activated the brain regions that are also associated with the concrete task and thus enhance performance. Similarly, if a mental training is able to activate areas that are associated with cognitive performance, the performance itself can be improved. However, the mental practice should be regularly trained for maintaining effects (25). In our study, participants received only one session of mental training. Furthermore, several cognitive abilities are needed for assessing attention performance in a specific task like the d2R that was used in our study, for example performance speed, accuracy, inhibition of distractors etc. (22). It might be possible that our mental training was not able to activate the specific abilities that were necessary for the attention task that we measured. Our training, which consisted of suggestions that might indirectly influence their performance, did not include any mental practice of the specific attention task. However, some higher order cognitions, as for example self-efficacy (27) or other meta-cognitive aspects like self-regulation or motivation (28), could have been more important for the cognitive outcome that we measured. Especially in student samples, perceived self-efficacy can influence the cognitive performance (29). The finding of another study, that verbal suggestions could enhance creativity, was explained by the idea that it was driven by intrinsic motivation and the belief in the own competence of the participant (30). If we transfer that explanation to our results, we could hypothesize that the participant's own motivation and belief in their performance had the biggest effect on their actual performance regardless of the information given by the investigator or the suggestions that were used in the mental training. Their own evaluation of the effectiveness of the training was consequently not based on external information but on their own intrinsic standards. The participants' motivation to improve or their perceived self-efficacy therefore influenced their performance the most. Our results are also in line with previous findings that it was rather the subjective evaluation than the objective performance that was influenced by verbal suggestions [see (7)]. The (almost significant) result that the cognitive performance improved more when subjects were given information about the type of training that was not congruent to the actual training they received, is extremely interesting within the previously discussed explanation. We interpret that result in the way that the participants' motivation to improve was even triggered more when receiving incongruent information.

We found that participants who rated the training as effective had higher hypnotic suggestibility than those who rated the training as ineffective. The effectiveness rating, however, was significantly influenced by the intervention they perceived they have received rather than the actual intervention condition that they received. This potential placebo effect implies that highly suggestible subjects might base their expectations on the owns appraisal instead of external information. We argue that the effect of enhancing cognitive performance was more pronounced in a subgroup of participants with high intrinsic motivation, high self-efficacy, and high suggestible ability. Similarly, patients with high suggestibility suffering from depression showed greater responses to suggestions and expectations regarding the effects and side effects they perceived together with taking an antidepressant medication (31). The ability to absorb in images, also known as hypnotic suggestibility [see (24)], might therefore mediate the effect of expectations on outcome [see also (31)]. However, the ability to absorb in our study had no impact on the cognitive performance itself but on the participants' own evaluation of the effectiveness of the treatment. This underlines the importance of tailoring interventions to some of the participants' characteristics or needs. Personalized medicine, also used in the psychiatric context, is based on that idea of optimizing the fit between patient characteristics and treatment choice and therefore enhancing treatment outcome and benefit [e.g., (32)]. Furthermore, interventions that are based on hypnotic verbal suggestions should have the goal of increasing the self-efficacy and the belief in the own competence (33). This idea can be underlined by the findings regarding the influence of non-specific/common factors on the outcome of psychotherapy (34).

Placebo effects on cognitive performances were found when participants suffer from mild cognitive or attentional deficits as for example in some nicotine-smokers that were deprived before (35). Some patients with Major Depression also suffer from a cognitive impairment [see (36)], and attention or concentration problems are also included in the list of typical symptoms and criteria of depression. Mental trainings and other psychological interventions for enhancing cognitive performance might even be more effective for these patients compared to healthy academically high performers like university students. This is in line with a study that found that older adults might also profit in their cognitive performance after receiving some kind of cognitive training (37). Future studies should evaluate the effects of a mental training that focuses on cognitive enhancement especially in patients with Major Depression. Within this context, the influence of placebo effects on the cognitive performance should also be investigated.

## Limitations

There are several factors that limit the generalizability of the current study.

One limitation of the current study is that we did not conduct any pilot study to figure out if the mental training that we conceptualized was effective or not. We also did not obtain any feedback about the quality of the mental training from the participants. However, we have to note, that it was not our goal to evaluate the mental training. In contrast, we were more interested in differentiating the effects of direct suggestions and information that were given by the investigators compared to creating some images (within the mental training) that might indirectly influence the participants' performance. However, the mental training should have been evaluated in different samples regarding its effectiveness on enhancing cognitive performance. For this purpose, a full deceptive placebo design could be used. In summary, our mental training was not specifically effective for the cognitive performance in the student sample.

Second, our study sample was not representative. We measured a very young and highly educated student sample. Comparing the means of the present sample with the norms of the d2R, it was obvious that even at baseline the student sample showed an extremely high concentration performance [norms of the d2R at the age of 20–39: *M* = 158.6, *SD* = 29.4, see (22); current sample: *M* = 174.38, *SD* = 36.24]. This might be based on motivation differences regarding the assessment or some previous experience in the task [see also (22)].

We found differences in the cognitive performance of participants at baseline regarding the factor type of information. The differences were found at baseline where the instruction were not yet given to the subject. Thus, we might have created an investigator's effect. Even if the participants were randomly assigned to the experimental condition, the investigator was not blind regarding the intervention and information condition that the subjects received when interacting with him or her. Especially when the investigator knew the fact that the participant will be later told to receive the control condition, it might have been that he or she behaved in a different way when interacting with the participant that may have influenced and increased their performance. We wanted to avoid any influence of the investigator on the subject by using a standardized protocol for instructing the subjects. But maybe they were already influencing the subjects' performance unconsciously or via indirect communication. In sum, a potential investigators' allegiance effect may have confounded the results [see also (38)]. Future studies should either avoid investigator effects by blinding the investigator who is interacting with the participants regarding the type of training they receive. Another possibility could also be to directly manipulate and vary of some aspects of the contact with the participants. For example, placebo effects were enhanced when a practitioner contact was longer and focused more on the nature and history of symptom assessment compared to a relationship with only limited contact (39) and a warm empathic contact with a clinician could even result in subjective and objective ratings of improvement of cold duration and severity (40).

## Conclusions

The participants' own evaluation of the effectiveness of the training was most probably driven by their own performance in the first and second trial of the task or by their own motivation to perform. The own experiences and ratings were subsequently more important for their cognitive performance than the efficacy of a specific training or information about the training they receive. This finding underlines the relevance of enhancing the self-efficacy in situations where cognitive attention processes are important and of individually tailoring psychological trainings or interventions accordingly. The relevance of mental trainings for people with psychological disorders with a mild cognitive impairment as for example in patients with mild to moderate Major Depression Episodes should be investigated in future studies. Within this context, especially the participant's belief in the efficacy of a specific treatment should enhance their actual treatment response. The ability to absorb in images, also known as hypnotic suggestibility [see (24)], might mediate the effect of expectations on outcome and should be investigated in future psychotherapy studies.

## Data availability statement

The raw data supporting the conclusions of this manuscript will be made available by the authors, without undue reservation, to any qualified researcher.

## Author contributions

Both authors contributed to the conception and design of the study. KF wrote the first draft of the manuscript. KF and DW conducted the statistical analysis. Both authors approved the submitted version.

### Conflict of interest statement

The authors declare that the research was conducted in the absence of any commercial or financial relationships that could be construed as a potential conflict of interest.
